# Institutional work to maintain, repair, and improve the regulatory regime: How actors respond to external challenges in the public supervision of ongoing clinical trials in the Netherlands

**DOI:** 10.1371/journal.pone.0236545

**Published:** 2020-07-31

**Authors:** Jacqueline C. F. van Oijen, Iris Wallenburg, Roland Bal, Kor J. Grit

**Affiliations:** Erasmus School of Health Policy & Management, Erasmus University Rotterdam, Rotterdam, The Netherlands; Amsterdam Universitair Medische Centra, NETHERLANDS

## Abstract

**Background:**

National regulatory regimes for supervising ongoing clinical trials are affected by external challenges, both international, such as harmonization of EU legislation, and national, such as critical reviews of incidents. This study examines how supervisory bodies dealing with ongoing trials respond to external challenges of the past two decades and engage in institutional work to maintain, repair, or improve the Dutch regulatory regime.

**Methods:**

International and national regulatory documents were analyzed and interviews (*n* = 27) were conducted with various actors, including public supervisory bodies, hospital staff, and boards of directors.

**Findings:**

In the Netherlands, EU harmonization directed at centralizing and coordinating the regulatory regime for good clinical trial practice in Member States has paradoxically led to further fragmentation. The resulting ambiguity and inefficiency remained largely unresolved until a serious incident in a university hospital became a catalyst to clarify both the interconnected responsibilities and working relationships of various supervisory bodies. New legislation and regulatory methods were implemented, and actors outside the legislative framework became active in the field in order to strengthen supervision of ongoing trials, further multiplying yet also aligning with existing regulatory regimes.

**Conclusions:**

Public supervision of ongoing trials is fragmented in the Netherlands because the responsibilities and resources are unevenly distributed. In countries like the Netherlands, public supervisory bodies must do a great deal of institutional work to align with new EU regulations and still safeguard their traditional regulatory mechanisms that protect human safety. However, national regulatory traditions also offer new opportunities to strengthen the quality assurance of clinical trials.

## § 1. Introduction

Clinical trial practice is highly regulated [1]. While it is accepted that some risk is inevitable, regulatory regimes are intended to reduce risk to a minimum [2]. To secure protection of human subjects and data validity and integrity, clinical trial practice is enforced by legislation and the institutionalized practice of supervision, together constituting the regulatory regime. Supervisory bodies must simultaneously change and maintain their regulatory regime in response to challenges such as critical reviews of national legislation or severe incidents.

In recent years, various attempts have been made to harmonize legislation and regulation of ongoing clinical trials within the European Union (EU). EU harmonization creates a massive ongoing challenge for Member States to align international regulation with national law and supervision practices, with various national attempts taking place over time [3]. The Netherlands in particular provides an interesting case to analyze the work required to achieve alignment with new EU rules and national regulatory regimes, since the traditional Dutch system differs significantly from the EU framework. Moreover, this country was confronted with a severe incident, the Propatria case, which raised a lot of media coverage. This paper focuses on the supervision of ongoing clinical trials in the Netherlands, as this area is less clearly regulated than the approval phase.

This paper uses an institutional theoretical framework, adopting the framework of Hood, Rothstein, and Baldwin [4] to explore how the regulatory regimes of clinical trials work and understand the forces that shape them. The concept of institutional work elucidates the dynamic interplay between actors and institutions [5], and focuses on how actors deal with external challenges, how they enact and adapt their everyday regulatory practices, and how they cooperate when reacting to external challenges. It enables us to examine in-depth how developments in EU legislation, and pressure from regulatory reviews and incidents, triggered changes in the Dutch regulatory regime, creating incongruity between legislation and actual practice in the supervision of ongoing trials.

The research question guiding this paper is: How do supervisory bodies in the public supervision of ongoing clinical trials in the Netherlands respond to the external challenges of the past two decades and engage in institutional work to maintain, repair, and improve the Dutch regulatory regime?

We detail how supervisory bodies faced with external pressures undertake institutional work to both change and preserve their institutions. We look at how alignment between the various supervisory bodies comes about, where frictions occur, how these are handled, and what kind of work is needed. In our case study, these challenges, and subsequently their dynamics and frictions, not only call for repair or maintenance work, but also create new space to stimulate action for improvement.

The paper is constructed as follows. The next section focuses on how the risk regulation framework and institutional work theory can help us study change and continuity in the institutional regulation regime of ongoing trials. Section 3 explains the regulatory regime of clinical trials in the Netherlands. Section 4 describes the research methods, while section 5 presents the results. Finally, section 6 presents the discussion, describing the impact of our results on both theory and regulatory practice and ending with conclusions. We believe that the mechanisms the Dutch public supervisory bodies use to deal with external challenges are relevant to other countries and domains, as any national supervisory body has to respond to these challenges within their own traditions.

## § 2. Theory

### A risk regulation regime framework

Hood, Rothstein, and Baldwin (2001) define risk regulation regimes as "the complex of institutional geography, rules, practice and animating ideas that are associated with the regulation of a particular risk or hazard" [4]. Overall, risk-based regulation aims to set standards, collect information, and influence and change behavior. Risk regulation regimes are based on three features. First, regimes are seen as systems, as sets of related, interacting parts. They are interested in both the activities of front-line people and the standard-setters and policy-makers at the center of government, as well as the relationship, if any, between the two. Second, regulation regimes have some degree of continuity over time. Third, because of the system-based approach, regimes are conceived as "relatively bounded systems that can be specified at different levels of breadth" [4]. Consequently, it is important to specify carefully which level of regime is being analyzed and the kind of risk the regime addresses.

### Institutional work

The concept of a regulatory regime stresses that "institutions matter" [6]. Institutions are commonly defined as systems of prevalent, established rules that structure social interactions [7]. They provide a degree of stability and have an important regulatory function in society [8]. This does not mean that institutions cannot change. This article uses the concept of institutional work to consider both stability and change. This concept can help analyze how regulatory actors not only respond passively to external challenges but also actively engage in three types of institutional work: the creation, maintenance, and disruption of institutions [5, 9]. Creation work involves establishing rules and constructing rewards and sanctions that enforce these rules. Maintenance work entails supporting, repairing, and recreating social mechanisms that ensure compliance with existing institutional norms. It seeks to ensure conformance with rules and systems and reproduce prevailing norms and belief systems. Disruption work involves attacking or undermining the mechanisms that lead actors to comply with institutions [5]. Institutional work theory thus draws attention to a relatively overlooked subject in mainstream institutional theory: the lived experiences of organizational actors [10, 11]. It suggests studying actions in a day-to-day setting to focus on local, creative, incremental practices and processes rather than on outcomes to gain an understanding of how institutions evolve.

The notion of institutional work is of particular interest to our investigation of the public supervision of clinical trials in the Netherlands for three reasons. First, it recognizes public supervisory bodies as embedded agents who are not merely executors of regulation, but whose activities contribute to shaping institutional regulatory regimes. Second, implied in the notion of institutional work is the idea of effort in the face of resistance or challenge. Institutional work is considered true "work," as it involves challenging and negotiating existing rules, practices, and beliefs that may be in opposition [12]. Third, it recognizes the distributed, pluralistic nature of change in the regulatory regime, where regulatory bodies interact with a wide spectrum of actors, none of whom have complete control or oversight, hence underscoring the aspect of ongoing regulatory uncertainty [13]. While one public supervisory body may strive to disrupt institutional arrangements and create new ones, others may strive to maintain those that appear to favor them. Hence, the theory of institutional work allows us to observe the immediate effects of new regulations and incidents and the mundane practices of institutional repair and maintenance work that they set in motion.

EU harmonization attempts, incidents, and other triggers may reveal a misfit between legal regulation and the daily practice of supervision of ongoing trials. Actors manage, exploit, and adjust their actions to the ambiguity, pluralism, and contradiction in regulatory regimes [14].

In this study, we seek to explore the institutional work of three Dutch public supervisory bodies in the regulatory regime of ongoing trials, tracing the effects of external challenges on their working methods and relationships. Before turning to our findings, we will first explain the institutional regulatory regime of clinical trials in the Netherlands.

## § 3. The regulatory regime of clinical trials in the Netherlands

The Netherlands has a decentralized structure of supervision, introduced in 1999 with the Medical Research Involving Human Subjects Act (WMO) [15, 16]. It stipulates the conditions permitting clinical research involving human subjects in the Netherlands and established a new supervisory body: the Central Committee on Research Involving Human Subjects (CCMO). Unlike other countries in the EU, in the Netherlands the assessment of research protocols is based on the historically institutionalized notion that science and ethics cannot be viewed separately; these two aspects come together in an integrated assessment procedure carried out by local medical research ethics committees (MRECs). This perspective, however, conflicts with the EU vision that the two aspects must be reviewed by separate bodies [17].

The WMO stipulates that a sponsor of a clinical trial with human subjects may not start the trial until an MREC has approved the research protocols. Most MRECs are linked to a university medical center (UMC) or one or more general hospitals, and a few work independently. MRECs are accredited and supervised by the CCMO, which can create new guidelines, for instance with regards to the required expertise of MREC members. Research proposals and their MREC assessments must be registered with the CCMO. The Health and Youth Care Inspectorate (IGJ), in turn, is responsible for verifying compliance with the WMO [15, Article 28] and for conducting inspections of clinical trials.

In 2004, the EU Clinical Trials Directive 2001/20/EC (EUCTD) was introduced [18]. The EUCTD aims to harmonize rules for clinical trials conducted across EU Member States. In the EU framework, supervision of clinical trials is the responsibility of Member States. Each Member State has sought alignment with the EUCTD based on their existing systems and traditions. The Dutch procedure of decentralized supervision deviates from the centralized, separated assessment procedure that the EUCTD advocates, which is more in line with other EU countries' regulatory systems, such as that of the UK.

**Fig 1:**
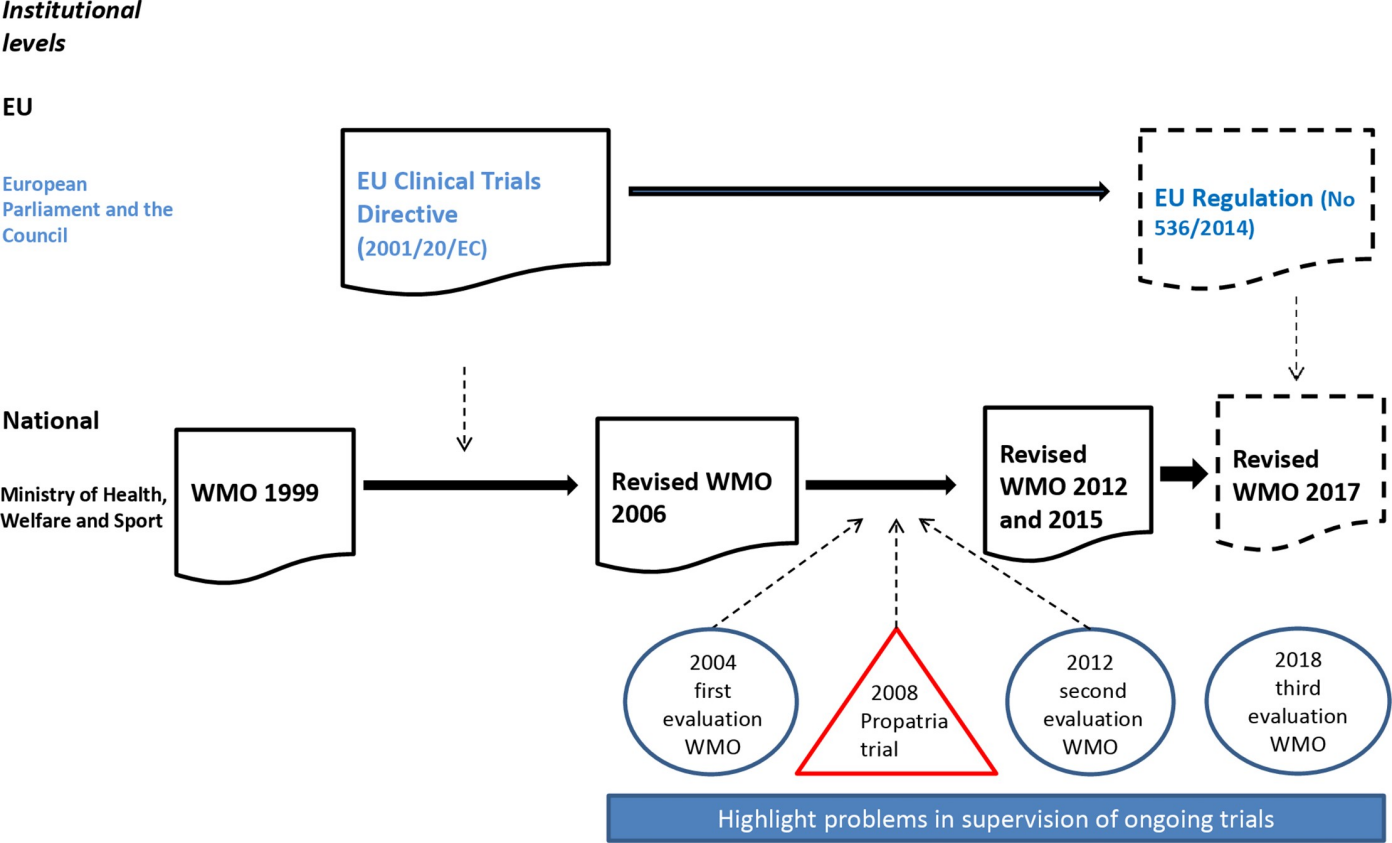
The regulatory regime in the Netherlands and the impetus to focus on supervision of ongoing trials.

The Dutch government decided to implement the EUCTD by modifying the WMO (2006) [19]. It created a special section for clinical trials that meets the requirements of the EUCTD [19, Article 13]. The EUCTD requires a clinical trial to be approved separately by a competent authority assessing the medical and scientific aspects of a protocol and an ethics committee verifying the primary ethical concerns [18, Article 2]. The Dutch government installed a dual review process, which continues the established integrated assessment of protocols by MRECs and adds a marginal role for the CCMO to act as competent authority. Following this procedure, in cases where the CCMO acts as the reviewing committee, the Ministry of Health, Welfare and Sport is the competent authority [19, Article 13-i and -j]. In a more general sense, the IGJ also fulfills the role of competent authority. This set-up is quite different in other European countries that have only one competent authority, such as the Medicines and Healthcare products Regulatory Agency, the authority responsible for clinical trial approval, oversight, and inspections in the UK. Whereas the UK has one supervisory body for ongoing trials, the Dutch have these three main bodies, resulting in a fragmented regulatory regime [17] (see Table 1 for an overview of the responsibilities of the Dutch public supervisory bodies the IGJ, CCMO, and MRECs; see Fig 1 for the historical development of the regulatory regime).

**Table 1:** Responsibilities of Dutch supervisory bodies laid down in the WMO in 1999 and 2006 after implementing the EUCTD.

Supervisory body	Level	Responsibility	WMO 1999 [15]	Added responsibility	WMO 2006 [19]
IGJ	Centralized	Verifying compliance with the provisions laid down by the WMO and conducting inspections of clinical trials	Article 28	At the request of the CCMO or Ministry of Health, verifying whether a clinical trial involving medicinal products is in accordance with current WMO	Article 13j
CCMO	Centralized	Regulating the accreditation of MRECs and overseeing their operations	Articles 16 and 24	Acting as competent authority if an MREC is the reviewing committee	Article 13i
MRECs*	Decentralized	Assessing and approving research protocols	Article 2	Receiving safety reports of ongoing trials involving medicinal products	Article 13o and 13p

* Important note: MRECs conduct many of the responsibilities of a competent authority, but are not regarded as a competent authority themselves.

Since 2000, the Dutch regulatory regime for assessing clinical trials has met several challenges. Reviews of national legislation and supervisory practices have exposed several weaknesses in the regulatory regime. The first evaluation of the WMO in 2004, prior to the introduction of the EUCTD, highlighted the unclear division of tasks between the IGJ and CCMO [20]. The second evaluation in 2012 revealed that responsibilities regarding the handling of serious adverse events (SAEs) were unclear [21]. The third evaluation in 2018 showed issues created by the complicated working relationships among the IGJ, CCMO, and MRECs [22]. Overall, the evaluation reports noted bottlenecks in the regulatory regime and task division between public supervisory bodies in the supervision of ongoing trials.

Besides EU harmonization and critical reviews of regulation, the Dutch regime is affected by incidents that attract public attention and act as catalysts [23], such as the Propatria trial in 2008, which was widely covered by the Dutch media. This investigator-initiated trial (IIT), a probiotic study of acute pancreatitis, was conducted in fifteen hospitals. As the sponsor, the UMC leading the study took responsibility for the initiation, management, and financing [18, Article 2]. Twenty-four patients in the probiotic group died of their disease, compared to nine patients in the placebo group (see Fig 1 for place in timeline).

The subsequent investigation conducted by the IGJ and CCMO, among others, highlighted several serious shortcomings in the design and execution of the research protocol, the information on side effects provided to the patients, and the reporting of SAEs―only two of the 33 deaths were reported immediately [24–26]. Furthermore, the Propatria report revealed that the hospital's board of directors failed to meet its responsibilities as sponsor in terms of the WMO. The safety of human subjects had been inadequately secured because several actors had not ensured that clear and efficient reporting procedures were in place [24]. The recommendations of the Propatria report thus fostered a focus on the roles and responsibilities of the MRECs as a supervisory body, and on the boards of hospitals as a sponsor. We expand on this in the results section, but first let us discuss our methods.

## § 4. Methods

### Research design

To gain insight into how and why changes in the regulatory regime of ongoing trials do or do not occur due to external challenges and how Dutch public supervisory bodies undertake institutional work to engage with these challenges and preserve their institutions, we conducted an exploratory qualitative study. First, to understand the chronology of changes to the regulation of clinical trials and the responses of public supervisory bodies, we studied documents on the Dutch situation, such as legal documents, annual reports of supervisory bodies, and previous research on the development of regulation regarding clinical trials (see S1 Table). The starting point of this document study was 1999, the year the WMO was launched and the CCMO was established. We use 2018 as the end point, as the consequences of the next round of harmonization, the European Clinical Trials Regulation No 536/2014 (ECTR) [27], became apparent then and the third evaluation of the WMO was published.

Second, we conducted semi-structured interviews to study the institutional work of actors involved in the public supervision of clinical trials. We interviewed inspectors from the IGJ (*n* = 9; one inspector three times); employees of the CCMO (*n* = 3; one employee twice) and the MRECs (*n* = 5); as well as the board and staff of hospitals (*n* = 10; one staff member twice). Interviews focused on working methods and mutual relationships (see topic lists in S1 Appendix) and took place between December 2013 and May 2018. With the permission of all respondents, the interviews were audio-recorded and transcribed in full. The interviews lasted between 40 and 90 minutes. The processed interview data were submitted to the respondents for member check. In the Netherlands this research requires no ethical approval.

### Data analysis

**Fig 2:**
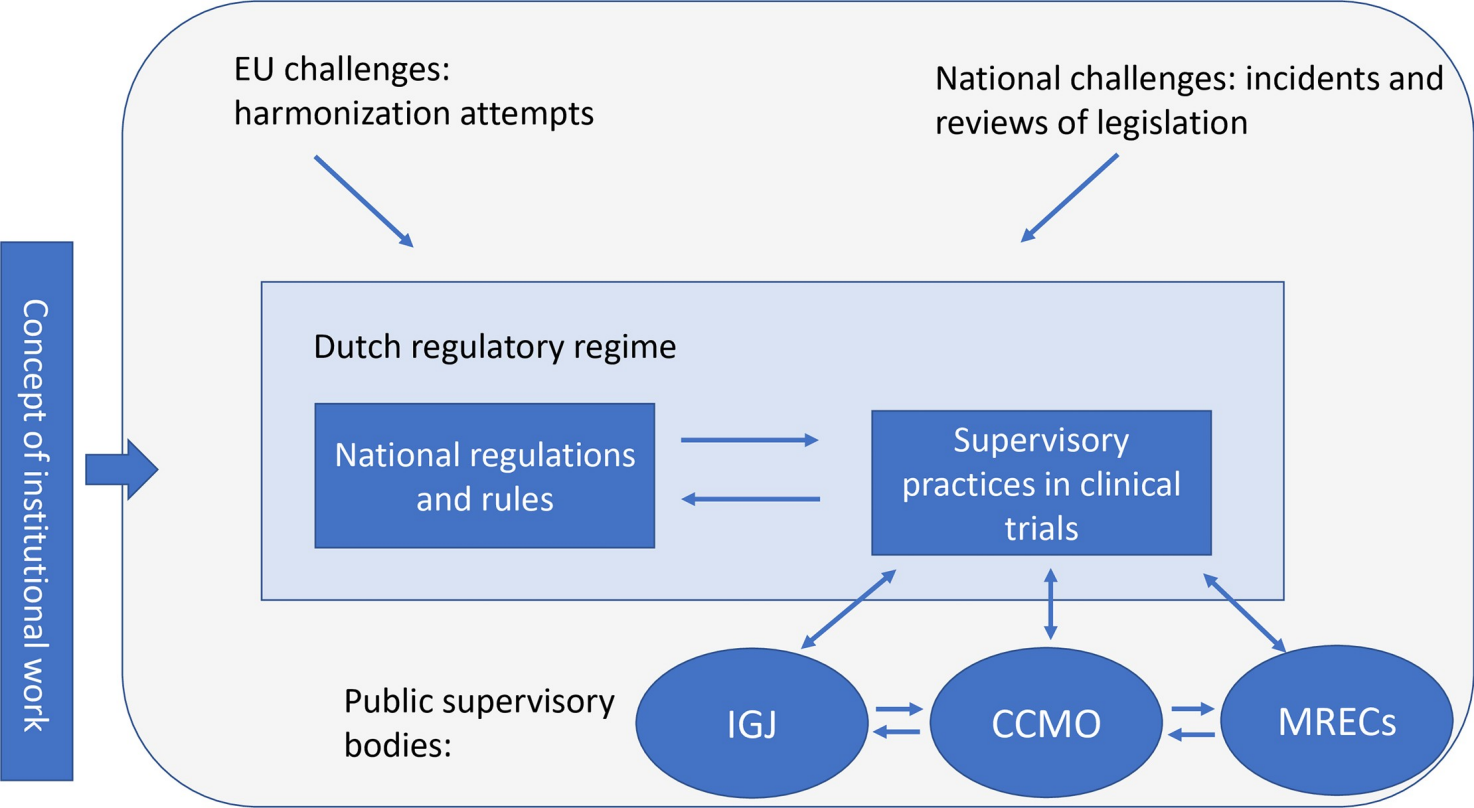
Overall scheme of the research focus.

Triangulating the results of the document analysis and the interviews enables us to develop an understanding of the external challenges Dutch public supervisory bodies faced in the past two decades and the kind of institutional work this required (see Fig 2).

When new issues emerged in interviews or in the news, we searched further for relevant documents, moving iteratively between our data (desk research and interviews) and the literature on risk regulation regimes and institutional work.

During data collection, we met regularly to analyze the data. Using inductive and deductive coding, based on regulatory regime and institutional work frameworks, we looked for relevant themes and the labels (codes) to index them. As forms of institutional work emerged, we debated the themes and codes until we reached a consensus. We identified three overarching themes that represented the supervisory bodies' responses to external challenges since 2000: (1) clarifying the division of roles and responsibilities in the supervision of ongoing trials, (2) dealing with the daily control of safety reports by MRECs, and (3) developing IIT inspections of hospitals as trial sites by the IGJ (see S2 Table).

## § 5. Results

Legal evolvement, critical evaluations, and the Propatria trial highlighted weaknesses in the supervision of ongoing trials. We analyzed how these challenges not only became effective catalysts for transforming processes in supervision, but also induced repair work to maintain the regulatory regime. This section shows how supervisory bodies such as the IGJ and CCMO do long-term institutional work, focusing on how they operate and endeavor to strengthen their own position (§ 5.1), and the position of other actors such as MRECs (§ 5.2) and hospital boards of directors (§ 5.3).

### § 5.1. Clarifying the division of roles and responsibilities in the supervision of ongoing trials

The national legislator's division of roles and responsibilities has influenced the relationship between the CCMO and IGJ. As pointed out above, the supervisory roles of both bodies have somewhat overlapped since the launch of the WMO in 1999 [28]. The WMO states that the IGJ must supervise the full scope of the WMO, which implies that it also supervises the entire system set for clinical trials subject to the WMO, including other supervisory bodies, which is a quite sensitive task:

Strictly, I think we [the IGJ] supervise it all, including the CCMO and the MRECs […]. But yes, no one likes that, so no one would ever admit it. (interview inspector 1 IGJ, 2014)

Discussion of each other's jurisdiction flared up in 2003, when the IGJ took the initiative to inspect several MRECs and the CCMO:

It's true, in the past we sometimes had differences of opinion with the IGJ: who supervises whom? And eventually, in 2003, the IGJ supervised MRECs, including us. But that didn't feel good, frankly. […] There's a lot open to interpretation: where does it [division of authority] begin and where does it end? (interview employee 1 CCMO, 2014)

Researchers involved in the first evaluation of the WMO in 2004 also observed this tension between the CCMO and IGJ [20]. The evaluation recommended focusing more on supervising ongoing trials and insisted on clarifying the responsibilities. In 2005, the Ministry of Health released a white paper that redefined the roles and responsibilities of the IGJ and the CCMO (see Table 2) to repair the regulatory regime. This form of maintenance work ensures compliance with existing regulation.

**Table 2:** Division of tasks between the IGJ and the CCMO [29].

Division of tasks	Supervisory body	Explained in more detail
1. Supervision aimed at quality improvement and harmonization of accredited MRECs	CCMO	§ 3 and § 5.2
2. Supervision of the CCMO: assessing whether the CCMO complies with the law	IGJ	§ 5.1
3. Supervision of compliance in practice (e.g. that the research is carried out according to the protocol, permission procedures have been adequately carried out, and there is an insurance policy)	IGJ	§ 5.2
4. Supervision as a result of an incident in ongoing research (e.g. the Propatria trial)	IGJ in cooperation with CCMO	§ 5.1–5.3

The IGJ received the explicit responsibility to supervise the CCMO. However, when we focus on this supervision, we see that this never really worked [22]. Beyond the aforementioned action taken in 2003, in practice the IGJ does not monitor the actions of the CCMO, allegedly because there has been no direct reason for the IGJ to take specific supervisory action in terms of monitoring risks or incidents.

After the Ministry allocated the roles and responsibilities, the IGJ and CMMO still needed to interpret their tasks. The IGJ's supervisory role means that if the results of an inspection necessitate a review of the MREC, the results are first passed to the CCMO. Therefore, the IGJ and CCMO need to communicate frequently to keep each other informed. At first, the communication was informal and less structured. After the WMO was introduced, however, they did not automatically exchange information. Based on its legal task, the CCMO manages a national registration system which records all ongoing studies assessed in the Netherlands. The IGJ has no access to this database, so if they want information on a particular study, they have to submit a request to the CCMO. In this relatively stable stage, the institutional work was aimed at maintaining the regulatory regime, organizing ad hoc information exchange so that both bodies could preserve their own roles and responsibilities.

The Propatria trial was a window of opportunity to discuss the relationship between the IGJ and CCMO because it forced both authorities to work together more closely. For the first time, the two investigated an incident together (see point 4 in Table 2). This led to a joint final report, together with the Netherlands Food and Consumer Product Safety Authority [24]. One CCMO employee recalls:

Over the years, we've realized that we just have to cooperate. The Propatria trial was the first time we really worked together on investigating an incident. It really instigated the cooperation with the IGJ, and everyone played their own part. It actually went very well, and so did the drafting of our joint report, which was based on the three separate reports from each authority. (interview employee 2 CCMO, 2018)

Subsequently, the IGJ and CCMO performed coordination work, as a form of creation work, organizing informal and formal consultations on both administrative and official levels. The purpose of the formal consultations is to discuss the practical implementation of matters that cover their legal tasks and responsibilities. For example, they agreed that the IGJ will inform the CCMO if it intends to visit an MREC during an inspection of an ongoing trial. The chosen division of tasks in the regulatory regime hence requires investment in cooperation between the CCMO and IGJ to ensure task coordination and the management of information [16]. This prompted the IGJ and CCMO to participate together in several EU groups working on the implementation of the ECTR [30,31]. In May 2018, they signed a protocol listing agreements on their mutual exchange of information and coordination [32]. This protocol can be seen as a form of maintenance work. It did not change the legal tasks and responsibilities of either organization, but was intended to prevent overlap or gaps in the supervision of ongoing clinical trials and in the enforcement of laws and regulations.

In short, although their roles and responsibilities were laid down in the WMO and later clarified in a white paper, they were not immediately taken over by the supervisory bodies. In practice, the lack of clarity caused tension, and information exchange was cumbersome. In terms of institutional work, coordination work done by the IGJ and CCMO was essential to respond to external challenges. The Propatria case created a policy window for them to organize their interconnected roles and responsibilities, which refined and strengthened their working relationship.

### § 5.2. Dealing with the daily control of safety reports by MRECs

The white paper of 2005 and the Propatria incident, however, did not altogether resolve the ambiguity surrounding the allocation of roles and responsibilities. The IGJ is responsible for conducting inspections of ongoing trials (see point 3 in Table 2). It does not assess research protocols beforehand, which is the task of MRECs, but depends on information provided by sponsors, researchers, and other competent authorities or supervisory bodies, such as annual safety reports, SAE reports, and notifications of unexpected but suspected adverse reactions. This information is primarily assessed by the MRECs, in line with the procedural requirements of the EUCTD.

How did the IGJ and MRECs attempt to compensate for the ambiguity surrounding their roles and tasks? To answer this question, we specifically look at the information flow and how the supervisory bodies process and assess the information received.

Legally, the MREC is tasked with assessing reported SAEs and other sponsor notifications from the standpoint of protecting human subjects [33]. However, we observed major differences between MRECs in the extent to which they are capable of meeting their obligations as stated in the WMO, resulting in major practice variation. Independent MRECs review many proposals for phase I and phase II studies, usually from contract research institutes and pharmaceutical companies that have more funding and pay for the MRECs' legal services. One independent MREC, with enough capacity and resources, closely follows and assesses safety reports, and even investigates the trial site to verify regulatory compliance, even if the legal status of its site report is doubtful:

We [independent MREC] visit trial sites ourselves at least once a year. [We] do a visitation as an MREC, to see if facilities and procedures are well organized. We pull out one of the ongoing studies at random, visit [it] and talk to one or more [of the] subjects. "What have you been told about this trial?" So, we act like an inspectorate. The status of our report is sometimes an opinion, sometimes a requirement. (interview chairman 1 MREC, 2014)

In contrast, hospital-based MRECs have limited funding and capacity, because they traditionally offer free services to trials executed in their "own" hospital, and capacity does not increase automatically with the increase in trials and submitted documents. These MRECs often find it impossible to fully process all safety reports and notifications, let alone verify their accuracy:

The detail level has gone up so much that it's a huge workload. Normally, I have this pile on Sundays [indicates stack of papers]. Now it can't come through the mail. Just compact discs. Yeah, it's really hopeless. You must take care you still pick out the essentials. (interview member of the CCMO and pharmacist-researcher at a top clinical teaching hospital, 2014)

Every week I get this extremely comprehensive set of SAEs where the researcher says that the SAE won't hinder the progress of the study. I believe them and [just] sign the papers blindly. It's correct, administratively. (interview chairman 2 MREC, 2014)

These quotes demonstrate the painstaking work involved in overseeing ongoing trials, but also reveal a lack of supervision by hospital-based MRECs. Our observations are in line with the second WMO review, which states that over half of MRECs found that supervision of ongoing trials was part of their task, but they usually did not have the workforce or financial means to execute it properly [21]. Consequently, a lapse in the supervision may occur when a sponsor reports an SAE to an MREC that cannot perform a substantive assessment. This can lead the sponsor to incorrectly conclude that the SAE is not a problem, or that a reassessment of the protocol is unneeded because the MREC has not undertaken any action.

One recommendation in the Propatria report was to report all SAEs in medical research with humans to the specific MREC responsible [24], which is already required for clinical trials with medicinal products (see Table 1). This recommendation prompted the legislator to repair the regulatory regime by mandating improvements to SAE reporting in the revised WMO of 2015, such as timely notification and ensuring that all relevant information about fatal or life-threatening SAEs is reported to the reviewing committee. Recurrently, the legislator did maintenance work to ensure compliance with the regulation. Additionally, the CCMO was legally obliged to report annually on the number of SAEs occurring in the preceding year. Previously, the CCMO had put great effort into gaining insight into SAEs and digitizing SAE reports. This creation work included reconstructing rules, property rights, and boundaries to gain access to SAE reports. Their new obligation gave insight into the amount of work MREC assessments of SAEs involved; 5808 [34] and 6103 [35] SAEs were reported in 2016 and 2017, respectively. Almost 5% of these SAEs had serious consequences for human subjects, leading to the termination or suspension of a trial [34–35].

In sum, MRECs deal differently with supervising ongoing trials due to funding, with MRECs in the not-for-profit sector having fewer resources available than those in the profit sector (e.g. pharmaceutical companies). This affects their ability to increase capacity when the workload grows. Over the years, the CCMO's creation work, done to gain a better grasp of SAE practice in ongoing trials, has led to a new legal responsibility for reporting annual numbers of SAEs. Despite all efforts, the division of tasks between the IGJ and MRECs is still unclear. The third WMO review suggests a new round of institutional work, involving the CCMO and hospital boards, to strengthen the position of the hospital-based MRECs in the regulatory regime [22].

### § 5.3. Developing IIT inspections of hospitals by the IGJ

The IGJ supervises the execution of all clinical trials in the Netherlands. To respond to and anticipate the results of the Propatria trial investigation, they began to focus increasingly on IITs.

The IGJ selected IITs as a theme to underscore the role and responsibility of hospital boards as sponsors of trials initiated by their organization. We observed major differences in the IGJ's working methods between the first and second round of inspections. In the first round, between 2014 and 2016, they carried out inspections targeting IITs of medical products at seven UMCs and two teaching hospitals. An inspector reflects on why IITs became the new focus:

We've been saying for years that in some studies it's not so clear that the sponsor [hospital] feels responsible; they should be, but are they really? And that's why the focus shifted to IITs. (interview inspector 2 IGJ, 2016)

A risk model for clinical trials of medical products was developed to make the sponsor's responsibilities transparent and place the hospital's board of directors into the position of sponsor. The legal framework was based on the International Council on Harmonization of Technical Requirements for Registration of Pharmaceuticals for Human Use Good Clinical Practice (ICH GCP) guideline [36], alongside the WMO. These two elements formed the basis for a detailed assessment of the hospital's practices. A four-day inspection of a pre-selected study examined the hospital's systems for the organization and execution of clinical trials. The IGJ shared the inspection results at various conferences. Here an inspector reflects on the hospital boards' attitude to their responsibility as sponsor:

So then you see the [hospital] boards generally do feel responsible, but the extent to which they ensure that a [quality] system gets implemented, well, that fluctuates. (interview inspector 2 IGJ, 2016)

The first round of results showed that general teaching hospitals were often involved in multicenter IITs. In 2016, the IGJ started a second round of inspections of IITs in teaching hospitals, anticipating that the level of quality assurance would differ because research is not a core business of teaching hospitals. Hence, the IGJ created a new database ranking hospitals by their number of studies and the table of contents of their quality assurance manuals. Using these criteria, ten teaching hospitals were selected for one-day inspections in 2016. The IGJ's creation work was to develop a new working method to obtain insight into actual safety practices while keeping the workload "doable". This method permitted quick scans of ongoing IITs rather than in-depth analysis of one IIT [37]. Each hospital was informed of the IGJ's focus on IITs and which departments they would visit. On the day of inspection the IGJ announced which IITs they wanted to review.

Okay, so we had to find a format to do it in fewer days—but that also means looking in less detail. It can't be otherwise. […] It's new, it's actually the first time that we're not just looking at clinical trials of medicinal products in our proactive supervision […] because […] supervising the WMO implementation doesn't just stop at clinical trials with medicinal products. Because of the ICH GCP, and the tools you have when you look at a study, most of the effort went into clinical trials, but that's different now too. (interview inspector 2 IGJ, 2016)

As the IGJ was no longer inspecting only clinical trials of medical products, the international ICH GCP became unusable. The IGJ sought new legislation which kept the focus on the responsibilities of the hospital boards. The Dutch Healthcare Quality, Complaints and Disputes Act of 2016 [38], developed for healthcare in general, formed the basis for its inspections. The IGJ's creation work incorporated altering the boundaries of regulatory systems and interweaving two regulatory regimes; hospital boards are now responsible for having a quality system available for clinical trials.

It is important to note that two of the teaching hospitals involved in the first round of inspections shared their critical findings and experiences with other teaching hospitals through the Association of Top Clinical Teaching Hospitals (STZ), an association of 26 teaching hospitals. This created a sense of urgency among other teaching hospitals, and prompted the STZ to undertake further supportive action. A staff member of one of these teaching hospitals explains:

Based on our inspection experience, we drew up a document called "Lessons learned". We first discussed this document internally and then with the STZ. It created a flywheel effect and, for example, led to adjustments to 33 STZ standard operating procedures. I think sharing is one of the strengths of the STZ. (interview staff member teaching hospital, 2014)

The STZ's creation work focused on examining "best practices" in top clinical teaching hospitals to create standard operating procedures, which hospitals can use to supplement their quality assurance manuals. This led to an upgraded level of quality assurance. The IGJ was pleasantly surprised to see this learning curve:

So, actually, it's nice because these hospitals had three years to pick it up. And of course it's also because the teaching hospitals were so open with the other teaching hospitals about what they'd gone through [in the first round of inspections after the Propatria case] and what they'd learned from the inspections and to share that with the others. That's where it starts naturally. (interview inspector 2 IGJ, 2016)

The IGJ halted their inspections after visiting eight of the planned ten teaching hospitals, because the same results and recommendations were evident in every hospital. This was made possible because, on the basis of the perceived sharing culture in the STZ, regarded as a serious partner in research, the IGJ presumed that the inspected hospitals would share their results with one another.

The inspectors shared their methods and the results of the inspections with the STZ at various meetings. This quote reveals the boards of directors' growing awareness, especially in teaching hospitals, of their tasks as sponsors of IITs:

It was so nice […], you really saw the penny drop. That someone said: "So, as a member of the board of directors, I'm responsible for monitoring the multicenter research that we do in other hospitals?" […] "Yes, that's right. And how you organize that—you can talk about that. You're responsible for it." (interview inspector 2 IGJ, 2017)

This came about through the IGJ's institutional work and by including the STZ in their fieldwork.

To sum up, the focus of the IGJ inspections shifted to IITs because of the Propatria trial. This shift involved positioning work by the IGJ, as a form of creation work. Subsequently, they created a context in which "new" actors outside of the public supervisory bodies, the hospital boards, were mobilized to take up their self-regulatory role [39]. The IGJ adopted a framework from a domain outside clinical trials that had to do with regulation of quality of care. Recasting the regulatory regime for hospitals was further stimulated by the Propatria case, underscoring teaching hospitals' interest in the quality assurance of research. Consequently, teaching hospitals could present themselves as research actors, something that used to be a privilege of UMCs.

## § 6. Discussion

The purpose of this paper is to examine how supervisory bodies in the public supervision of ongoing trials in the Netherlands respond to external challenges, and engage in institutional work to maintain, repair, and improve the regulatory regime for the safety of clinical trials. The paper shows that changes in (inter)national law and severe incidents in research practice created a window of opportunity for institutional work to both change and protect the regulatory regime. Our findings demonstrate that institutional work is a continuous endeavor at the level of regulatory regimes. Ambiguity sometimes complicated finding the right terms for institutional work. When done by a public supervisory body, institutional work can be considered creation work, changing the regulatory regime, but at the same time it can be regarded as protecting the existing regime. These become interconnected as public supervisory bodies respond to the threats to their status quo and the challenges that create new opportunities. In fact, public supervisory bodies must constantly adapt to external challenges in order to stay the same.

Paradoxically, this research shows that the EU policy of harmonization led to even more fragmentation in the Dutch regulatory regime. Implementing the EUCTD left the decentralized supervision structure in place, whereas the EUCTD stipulated a centralized system. In practice, the IGJ, CCMO, and MRECs needed clarity on who was responsible and accountable for what in the public supervision of ongoing trials. The Propatria trial became a catalyst for the IGJ and CCMO to perform institutional work and act more constructively, and it let teaching hospitals present themselves as research institutes. However, it left unclarities in place, such as the division of tasks between the IGJ and MRECs, especially in the supervision of ongoing research. The layered regulatory regime implies that public supervisory bodies also monitor the public tasks of other supervisory bodies: the CCMO supervises MRECs, while the IGJ supervises all the involved parties. However, between the IGJ and CCMO, the role of "supervising supervision" still needs clarification. Nowadays, the IGJ is advised to maintain a position such that it can supervise the CCMO [22]. In terms of institutional work, these examples show that not all issues can be resolved by public supervisory bodies, due to their historically rooted dominance and interests and the fact that these issues lie beyond their primary roles and capacities.

Overall, institutional theory offers conceptual tools for analyzing the work needed to make regulatory regimes productive. In countries like the Netherlands, with a different tradition from the EU regulatory framework, public supervisory bodies must carry out a great deal of institutional work to align with EU regulations. By focusing on the dynamic interplay between three supervisory bodies in the past two decades, this study contributes to the literature on the relational features of institutional work in two ways. First, our findings demonstrate that institutional work is needed at the level of the regulatory regime because the interplay between evolving regulations and external challenges creates certain dynamics and frictions. Public supervisory bodies continuously need to align with these dynamics while safeguarding existing effective regulatory mechanisms. Second, our findings reveal how public supervisory bodies deal with the external challenges presented by EU harmonization attempts and exposed weaknesses in the regulatory regime. The weaknesses highlighted in the supervision of ongoing IITs, reflected by one adverse incident, became an especially effective catalyst for maintaining and repairing the regulatory regime of the public supervision of ongoing trials. Although other forms of institutional work could be referenced, as the literature has discerned many categories and labels, the institutional work referred to in our case study dealt particularly with coordination work leading to improvements in information sharing, and positioning work to repair and maintain existing institutions. Our case study showed the importance of positioning work, meaning the mobilization and positioning of actors to assume specific roles or do new things, such as bringing actors from outside the legislative framework, in this case hospital boards and the STZ, onto the playing field. This creates new opportunities to strengthen the quality assurance of clinical trials in hospitals.

One limitation of our study is that it is based on a single case in the Netherlands. However, investigating mechanisms like institutional work requires very detailed data collection to link theory to empirical work [5]. Triangulation is used as leverage and, in our case study, involves data collected from different places, sources, times, and levels of analysis, and by different methods, such as interviewing stakeholders, analyzing documents, and composing historical descriptions [40]. Particularly, we were interested in the underlying mechanisms that allow regulatory regimes to adapt, given external and internal challenges. By sharing our research findings with several colleagues, from different fields of expertise, we tried to draw valid inferences. Overall, the depth of our study came at the expense of its width.

In conclusion, external challenges like attempts at EU harmonization can be complicated when Member States have divergent regulatory regimes. Harmonization requires more than "just implementing new rules." It requires institutional work by Member States to align existing regulatory regimes with new rules and balance between institutional change and preservation. Given the rise of increasingly international, multi-sited research, such work remains important to safeguard patient safety and data integrity. Zooming in on the supervision of trials in the Netherlands, we observed how EU harmonization attempts created tension in the Dutch regulatory regime and supervision practices. Institutional work is needed to resolve this tension, but new problems may arise from the solutions.

While our longitudinal study focused on the consequences of the EUCTD in the regulatory regime, Member States have prepared to implement the next round of harmonization, the ECTR, which will change the process for starting clinical trials in Europe yet again in 2020 [41] and replace the EUCTD and the national legislation created to implement it [42]. As a result, the Netherlands has modified the WMO (2017) to meet ECTR requirements and the CCMO has established a National Clinical Trial Office to offer administrative support to MRECs involved in the assessment of multinational studies in the Netherlands [43]. No end to institutional work is to be expected, given the ongoing adjustments to the existing regulatory regime needed to meet new EU requirements. This paper lays the conceptual and empirical groundwork for studying this kind of work.

## Supporting information

S1 Table Details of evidence used in this study. (DOCX)

S2 Table Themes and their related codes. (DOCX)

S1 Appendix Topic lists. (DOCX)

## Abbreviations

CCMO: Central Committee on Research Involving Human Subjects
ECTR: European Clinical Trials Regulation No 536/2014
EU: European Union
EUCTD: EU Clinical Trials Directive 2001/20/EC
MREC: medical research ethics committee
IGJ: Health and Youth Care Inspectorate
ICH GCP: International Council on Harmonization of Technical Requirements for Registration of Pharmaceuticals for Human Use Good Clinical Practice
IIT: investigator-initiated trial
SAE: serious adverse event
STZ: Association of Top Clinical Teaching Hospitals
UK: United Kingdom
UMC: university medical center
WMO: Medical Research Involving Human Subjects Act

## References

[1] Keating P, Cambrosio A. Cancer on Trial: Oncology as a New Style of Practice. Chicago: University of Chicago Press; 2012.

[2] Seiler H. Harmonised Risk Based Regulation-a legal viewpoint. Safety science. 2002;40(1–4): 31–49.

[3] McMahon AD, Conway DI, MacDonald TM, McInnes GT. The Unintended Consequences of Clinical Trials Regulations. PLoS Med. 2009; 6(11): e1000131 10.1371/journal.pmed.1000131 PMC2768793 19918557

[4] Hood C, Rothstein H, Baldwin R. The government of risk: Understanding risk regulation regimes. Oxford: Oxford University Press; 2001.

[5] Lawrence TB, Suddaby R. Institutions and institutional work In Clegg SR, Hardy C, Lawrence TB, Nord WR, editors. Handbook of organization studies. London: SAGE; 2006 pp. 215–254.

[6] Windholz E.L. (2018) Govering though Regulation Public Policy, Regulation and the Law. Routledge, New York.

[7] Hodgson GM. What Are Institutions? Journal of Economic Issues. 2006; 40:1: 1–25, 10.1080/00213624.2006.11506879

[8] Beunen R, Patterson J. Analysing institutional change in environmental governance: exploring the concept of ‘institutional work'. Journal of Environmental Planning and Management. 2016: 1–18. 10.1080/09640568.2016.1257423

[9] Bochove M, Oldenhof L. Institutional Work in Changing Public Service Organizations: The Interplay Between Professionalization Strategies of Non-Elite Actors. Administration & Society. 2018: 1–27. 10.1177/0095399718786880

[10] Lawrence TB, Leba B, Zilber TB. Institutional Work: Current Research, New Directions and Over looked Issues. Organization Studies. 2013;34(8): 1023–1033.

[11] Lawrence TB, Suddaby R, Leca B. Institutional work: Refocusing institutional studies of organization. Journal of Management Inquiry. 2011;20: 52–58.

[12] Wallenburg I, Quartz J, Bal R. Making hospitals governable. Performativity and institutional work in ranking practices. Administration & Society 2016: 1–27. 10.1177/0095399716680054

[13] Mills R, Koliba C. The challenge of accountability in complex regulatory networks: the case of Deepwater Horizon oil spill. Regulation & Governance. 2015;9: 77–91.

[14] Cloutier C, Denis J-L, Langley A, Lamothe L. Agency at the Managerial Interface: Public Sector Reform as Institutional Work. Journal of Public Administration Research And Theory 2016;2: 259–276. 10.1093/jopart/muv009

[15] Staatsblad van het Koninkrijk der Nederlanden. Wet van 26 februari 1998, houdende regelen inzake medisch-wetenschappelijk onderzoek met mensen (Wet medisch-wetenschappelijk onderzoek met mensen) [Medical Research Involving Human Subjects Act], Stb. 1998, 161.

[16] Centrale Commissie Mensgebonden Onderzoek (2009). Toezicht en toetsing in de toekomst. [Supervision and review in the future] Den Haag: Centrale Commissie Mensgebonden Onderzoek.

[17] Van Oijen JCF, Grit KJ, van de Bovenkamp HM, Bal RA. Effects of EU harmonization policies on national public supervision of clinical trials: A dynamic cycle of institutional change and institutional work. Health Policy. 2017;121: 971–977. 10.1016/j.healthpol.2017.06.008 28733068

[18] European Parliament and of Council of the European Union. Directive 2001/20/EC of the European Parliament and the Council of 4 April 2001 on the approximation of the laws, regulations and administrative provisions of the Member States relating to the implementation of good clinical practice in the conduct of clinical trials on medicinal products for human use. Official Journal of the European Communities 2001;L121(May): 34–44. 16276663

[19] Staatsblad van het Koninkrijk der Nederlanden. Wet van 30 november 2006 tot wijziging van diverse wetten op of in verband met het terrein van VWS, ten einde wetstechnische gebreken te herstellen en andere wijzigingen van ondergeschikte aard aan te brengen (Reparatiewet VWS 2006). Artikel XVII wijzigt de Wet medisch-wetenschappelijk onderzoek met mensen [Article XVII changes the Medical Research Involving Human Subjects Act]. Den Haag: Sdu Uitgevers; 2006, 644.

[20] Dute JCJ, Friele RD, Nys H, Op den Drink VAJ, Van Gils RCW, Eysink PED, et al Evaluatie Wet medisch-wetenschappelijk onderzoek met mensen [Evaluation of the Medical Research Involving Human Subjects Act]. Den Haag: ZonMw; 2004.

[21] Stukart MJ, Olsthoorn-Heim ETM, van de Vathorst S, van der Heide A, Tromp K, de Klerk C. Tweede evaluatie Wet medisch-wetenschappelijk onderzoek met mensen [Second evaluation of the Medical Research Involving Human Subjects Act]. Den Haag: ZonMw; 2012.

[22] Ploem MC, Woestenburg NOM, Floor T, Van de Vathorst S, Geertsema B, Legemaate J, et al Derde evaluatie Wet medisch-wetenschappelijk onderzoek met mensen [Third evaluation of the Medical Research Involving Human Subjects Act]. Den Haag: ZonMw; 2018.

[23] Bozeman B, Anderson D. Public Policy and the Origins of Bureaucratic Red Tape: Implications of the Stanford Yacht Scandal. Administration and Society. 2016 8 1;48(6): 736–759. 10.1177/0095399714541265

[24] Inspectie voor de Gezondheidszorg, Centrale Commissie Mensgebonden Onderzoek en Voedsel en Waren Autoriteit. Onderzoek naar de Propatria-studie: Lessen voor het medisch-wetenschappelijk onderzoek met mensen in Nederland [Research tot the Propatria study: Lessons for the medical research involving human subject in the Netherlands]. Den Haag: Inspectie voor de Gezondheidszorg, Centrale Commissie Mensgebonden Onderzoek en Voedsel en Waren Autoriteit; 2009.

[25] Zaat J, De Leeuw P. IGZ-rapport over de Propatria studie. Lessen voor onderzoek. [IGJ-report on the Propatria study] Ned Tijdschr Geneeskd. 2009;153: B520. 20051171

[26] Sciencemag.org. Report slams deadly Dutch probiotic study. Available from: http://www.sciencemag.org/news/2009/12/report-slams-deadly-dutch-probiotic-study

[27] European Parliament and of the Council of the European Union. Regulation (EU) No 536/2014 of the European Parliament and of the Council of 16 April 2014 on clinical trials on medicinal products for human use, and repealing Directive /20/EC. Official Journal of the European Union 2001;L158(May): 1–76.

[28] Aartsen JGM. CCMO geïnstalleerd door de Minister van VWS. Graadmeter. 1999; 15(2): 3–5.

[29] Tweede Kamer der Staten Generaal. Evaluatie Wet medisch-wetenschappelijk onderzoek met mensen. Brief van de staatssecretaris van Volksgezondheid, Welzijn en Sport [Evaluation of the Medical Research Involving Human Subjects Act. Letter of the State Secretary for Health, Welfare and Sport]. Vergadering 2004–2005, 29963, 2: 8–9.

[30] Centrale Commissie Mensgebonden Onderzoek. Jaarverslag 2008 [Annual report 2008]. Den Haag, Centrale Commissie Mensgebonden Onderzoek; 2009.

[31] Inspectie voor de Gezondheidszorg. Jaarbeeld 2016 [Annual Review 2016]. Den Haag: Inspectie voor de Gezondheidszorg.

[32] Staatscourant. Samenwerkingsprotocol tussen de Centrale Commissie Mensgebonden Onderzoek en de Inspectie Gezondheidszorg en Jeugd [Cooperation protocol between the Central Committee on Research Involving Human Subjects and the Health and Youth Care Inspectorate], nr. 37731, 9 juli 2018.

[33] Tweede Kamer, vergaderjaar 2014–2015, 33 646, nr. 10.

[34] Centrale Commissie Mensgebonden Onderzoek. Jaarverslag 2016 [Annual report 2016]. Den Haag, Centrale Commissie Mensgebonden Onderzoek; 2017.

[35] Centrale Commissie Mensgebonden Onderzoek. Jaarverslag 2017 [Annual report 2017]. Den Haag, Centrale Commissie Mensgebonden Onderzoek; 2018.

[36] International Council on Harmonization of Technical Requirements for Registration of Pharmaceuticals for Human Use (ICH). ICH harmonized tripartite guideline. Guideline for good clinical practice: consolidated guideline; 1996. E6(R1), 10 June.

[37] Grit KJ, van Oijen JCF. Toezicht op het medisch-wetenschappelijk onderzoek met mensen: het in kaart brengen van een multi-centered speelveld [Supervision of medical research involving human subjects: mapping a multi-centered playing field] Rotterdam: iBMG, Erasmus Universiteit Rotterdam; 2015.

[38] Staatsblad van het Koninkrijk der Nederlanden. Wet van 7 oktober 2015, houdende regels ter bevordering van de kwaliteit van zorg en de behandeling van klachten en geschillen in de zorg (Wet kwaliteit, klachten en geschillen zorg) [Healthcare Quality, Complaints and Disputes Act], 11-11-2015.

[39] van Erp J, Wallenburg I, Bal R. Performance regulation in a networked healthcare system: From cosmetic to institutionalized compliance. Public Administration. 2018: 10.1111/padm.12541

[40] Brady HE, Collier D. (Eds.). Rethinking social inquiry: Diverse tools, shared standards.2^nd^ ed Lanham, MD: Rowman & Littlefield Publishers; 2010.

[41] Tenti E, Simonetti G, Bochicchio MT, Martinelli G. Main changes in European clinical trials regulation (no 536/2014). Contemporary clinical trials communications. 2018;11: 99–101. 10.1016/j.conctc.2018.05.014 30003173 PMC6039537

[42] European Commission. Clinical trials. Available from: https://ec.europa.eu/health/human-use/clinical-trials_en

[43] Ministerie van Volksgezondheid, Welzijn en Sport, Dutch Clinical Trial Foundation, Centrale Commissie Mensgebonden Onderzoek. ECTR European Clinical Trial Regulation. Clinical Trials. 2018. Available from: https://dcrfonline.nl/nieuws/brochure-over-de-ectr-beschikbaar/

