# Supplementary material for: Institutional work to maintain, repair, and improve the regulatory regime: How actors respond to external challenges in the public supervision of ongoing clinical trials in the Netherlands
Source: PLoS One. 2020 Jul 31;15(7):e0236545. doi: 10.1371/journal.pone.0236545 (PMC7394415; doi:10.1371/journal.pone.0236545)
Supplement: S1 Table — (DOCX) [file pone.0236545.s001.docx]

**Table 2.** *Details of evidence used in this study*

| **Interviews** |
| --- |
| In-depth interviews (*n*=27) with nine inspectors, three employees of the CCMO, five employees of MRECs, and ten staff or board members of hospitals. Some respondents were interviewed multiple times. |
|  |
| **An overview of analyzed documents from 2000 to 2018 (if any)** |
| - CCMO press releases, annual reports, guidelines, or other publications |
| - IGJ annual reports |
| - IGJ press releases |
| - MRECs (NVMREC) 'Forum' |
| - IGJ presentations to external stakeholders from 2014 to 2018 |
| - - Presentation to Board of directors University Medical Centers |
| - - Presentation to Board of directors STZ |
| - - Presentation at meeting of the Dutch Clinical Research Foundation |
| - Reports on the Propatria study |
| - Press releases of the Dutch Ministry of Health |
| - EU regulation governing clinical trials |
| - Dutch regulation governing clinical trials or medical research |
| - Reviews of EU legislation (EUCTD) |
| - Reviews of Dutch national legislation (WMO) |
| - Public consultation paper "Assessment of the functioning of the 'Clinical Trials Directive' 2001/20/EC" (2009) |
| - Revision of the Clinical Trials Directive. Concept paper submitted for public consultation (2011) |
| - Transcripts of all debates in the Dutch parliament on the regulation of clinical trials |
| - Media reports relating the regulation of clinical trials and incidents |
| - Dutch journals where any reference to clinical trials or medical research were made |
