# Supplementary material for: Institutional work to maintain, repair, and improve the regulatory regime: How actors respond to external challenges in the public supervision of ongoing clinical trials in the Netherlands
Source: PLoS One. 2020 Jul 31;15(7):e0236545. doi: 10.1371/journal.pone.0236545 (PMC7394415; doi:10.1371/journal.pone.0236545)
Supplement: S2 Table — (DOCX) [file pone.0236545.s002.docx]

**Table 3.** *Themes and their related codes*

| **Themes** | **Codes** |
| --- | --- |
| Interconnection/interdependence of responsibilities and working relationships among three public supervisory bodies |  |
| Division of roles and responsibilities in the supervision of ongoing trials: problems with diverging interpretations of roles and responsibilities; options to streamline and accelerate the supervision of ongoing trials | Redefining roles and responsibilities; frictions; jurisdiction; tension; conflicts; alignment "supervision of supervision"; information flow; (in)formal consultations; coordination tasks working together in EU working groups; protocol  Institutional work: maintenance, creation, coordination |
| Daily control of safety reports: problems with current method of reporting SAEs; options for improvement | Ambiguity in roles and responsibilities; information flow; workforce; funding; redefining and digitalizing reporting process of SAEs  Institutional work: maintenance, creation |
| IGJ inspections of IITs in hospitals: options for a risk-based approach that adopts different regulatory frameworks depending on the type of supervised hospital (UMC or teaching hospital) | Focus on one or more IITs; working method model; legal framework; duration and scope of inspections; sharing inspection results; stimulating quality assurance and self-regulation of sponsors through the Association of Top Clinical Teaching Hospitals  Institutional work: creation, positioning |
