# Supplementary material for: Institutional work to maintain, repair, and improve the regulatory regime: How actors respond to external challenges in the public supervision of ongoing clinical trials in the Netherlands
Source: PLoS One. 2020 Jul 31;15(7):e0236545. doi: 10.1371/journal.pone.0236545 (PMC7394415; doi:10.1371/journal.pone.0236545)
Supplement: S1 Appendix — (DOCX) [file pone.0236545.s003.docx]

**Appendix 2: Topic lists**

*Supervisory bodies (IGJ, CCMO, MRECs)*

Subject/dimensions:

- Their internal organization, resources, and capacities
- Their working methods: procedures for core regulatory activities, involvement in national and international activities, enforcement powers, and accountability
- Experience with their regulatory activities
- Working relationship with other supervisory bodies or actors: information gathering and flow, coordination, and cooperation activities, if any
- Dealing with external challenges: responses to changes in regulations and incidents; effects on mutual exchange of information, cooperation, and coordination with other supervisory bodies or actors

*Staff and board of hospitals*

Subject/dimensions:

- Their experiences with the regulatory activities of the IGJ, MRECs and CCMO
- Dealing with external challenges: responses to changes in regulations and incidents; their effects
